# Is there a relationship between surgical proficiency and oncologic outcome of minimally invasive radical hysterectomy for early-stage cervical cancer?

**DOI:** 10.7150/ijms.82113

**Published:** 2023-02-27

**Authors:** Jung Hwan Ahn, Jisu Yun, Chae Young Yun, Ji Geun Yoo, Sung Jong Lee, Joo Hee Yoon, Dong Choon Park, Sang Il Kim

**Affiliations:** 1 Department of Obstetrics and Gynecology, Yonsei University Wonju College of Medicine, Wonju, Republic of Korea.; 2 Department of Obstetrics and Gynecology, St. Vincent's Hospital, College of Medicine, The Catholic University of Korea, Seoul, Republic of Korea.; 3 Department of Obstetrics and Gynecology, Daejeon St. Mary's Hospital, College of Medicine, The Catholic University of Korea, Seoul, Republic of Korea.; 4 Department of Obstetrics and Gynecology, Seoul St. Mary's Hospital, College of Medicine, The Catholic University of Korea, Seoul, Republic of Korea.

**Keywords:** cervical cancer, LACC trial, learning curve, surgical proficiency, minimally invasive surgery, MIS.

## Abstract

**Objective:** Investigate the relationship between surgical proficiency and oncological outcomes of minimally invasive surgery (MIS) in the treatment of early-stage cervical cancer.

**Methods:** This retrospective study included patients with cervical cancer stage IB1, IB2 who were treated with minimally invasive radical hysterectomy from January 2010 to Dec 2020. Patients were divided into two groups based on the year of surgery: phase 1 (from January 2010 to December 2015) and phase 2 (from January 2016 to December 2020). Oncologic outcomes were compared between the groups.

**Results:** In total, 142 patients were included in the final analysis. 73 and 69 patients underwent surgery in phase 1 (51.4%) and phase 2 (48.6%), respectively. Twelve recurrences (12/142, 8.5%) were observed in the entire cohort: ten (13.7%) in phase 1 and two (2.9%) in phase 2. The recurrence rate was significantly higher in phase 1 (p = 0.021). And the phase 1 group showed significantly shorter disease-free survival than the phase 2 group (p = 0.049). In the multivariate analysis, surgical proficiency, represented by the phase of operation, was the only significant predictor of disease-free survival (HR = 0.244, p = 0.042).

**Conclusions:** Surgical proficiency in MIS is a significant factor associated with the outcomes in early-stage cervical cancer. More favorable outcomes can be obtained after operating on a certain number of MIS cases.

## Introduction

According to Global Cancer Statistics 2020, the number of new cases of cervical cancer worldwide is estimated to be 604,127 with 341,831 deaths 1. Among female cancer patients, cervical cancer accounts for 6.5% of new cases and 7.7% of deaths 1. Although the incidence of cervical cancer is declining owing to vaccination programs, it remains one of the most common gynecologic malignancies worldwide.

The standard treatment for early-stage cervical cancer without a bulky mass is radical hysterectomy with pelvic lymphadenectomy 2. Minimally invasive surgery (MIS) for cervical cancer was first described in 1992 3, 4. Since then, MIS for cervical surgery has become more common, replacing open surgery 5. Numerous studies have compared MIS and open surgery and reported the advantages of MIS, including lower operative morbidity and fewer postoperative complications with similar outcomes 6-8.

However, in November 2018, the Laparoscopic Approach to Cervical Cancer (LACC) trial reported significantly inferior oncologic outcomes with MIS in comparison with open surgery 9. These results led to dramatic changes in real-world clinical practice. Consequently, the National Comprehensive Cancer Network (NCCN) and European Society of Gynecological Oncology (ESGO) changed their recommendations for early-stage cervical cancer 2, 10. The NCCN suggest open surgery as the standard and recommended approach to radical hysterectomy 2. After the publication of the results of the LACC trial, the number of procedures performed via MIS dramatically decreased 11.

However, the randomized controlled surgical trials conducted as a part of the LACC study also had some limitations 12. One of the controversies surrounding the LACC trial was regarding the variation in skills and surgical proficiency of the participating centers and surgeons 13. In the LACC trial, the surgeon proficiency criteria for MIS were only 10 cases and two unedited videos 9. However, many gynecologic oncologists considered these criteria insufficient to indicate proficiency in MIS. Moreover, Park et al. suggested that there may be a high probability that the radicality of surgery was not fully achieved through MIS in the LACC trial 14.

Several studies have reported the correlation between surgical proficiency and clinical outcomes 15 - 18. However, evidence regarding the association of surgical proficiency with survival outcomes in MIS remains insufficient. The objective of this study was to investigate the relationship between surgical proficiency and oncological outcomes of MIS in the treatment of early-stage cervical cancer.

## Materials and Methods

This retrospective cohort study was approved by the Institutional Review Board of the Catholic University of Korea (VC23RISI0039). The requirement for informed consent was waived owing to the retrospective nature of the study.

### Study population

From our institution's cancer registry, we reviewed the medical records of patients who underwent MIS for cervical cancer between January 2010 and December 2020 at St. Vincent Hospital, Catholic University of Korea. Patients with a preoperative diagnosis of cervical cancer of squamous cell, adenocarcinoma, or adenosquamous histologies were included. Using the revised 2018 FIGO staging system, 167 patients who received primary surgical treatment and had histologically confirmed stage IB1 and IB2 disease were initially included 19. All patients underwent type C radical hysterectomy according to the Querleu-Morrow classification 20. The exclusion criteria were as follows: (1) any histologic type other than squamous cell carcinoma, adenocarcinoma, or adenosquamous carcinoma; (2) radiation therapy or neoadjuvant chemotherapy prior to surgery; and (3) insufficient clinical and/or pathological data. After surgery, adjuvant radiotherapy was selectively implemented owing to the risk of recurrence according to the Sedlis criteria 21.

All surgical procedures were performed at a single hospital by two board-certified gynecological oncologists with similar levels of experience in MIS. All patients underwent either conventional laparoscopic radical hysterectomy (LRH) or robot-assisted laparoscopic radical hysterectomy (RRH). RRH was performed using the da Vinci Si or Xi Surgical System (Intuitive Surgical, Inc., CA, USA). Surgeon B did not use the uterine manipulator. Surgeon A used a uterine manipulator until December 2015 but then stopped using it owing to the possibility of tumor dissemination.

### Data collection

We collected information about clinical and pathological characteristics (age, 2018 FIGO stage, surgical approach, operating surgeon, histologic type, grade, tumor size, risk factors, and surgical outcomes) and adjuvant treatments. Tumor size was documented as the longest diameter based on histopathological findings. Recurrence was confirmed by clinical and imaging findings and pathology reports. To determine whether surgical proficiency may have contributed to survival outcomes, we divided the study period into two phases: phase 1 (from January 2010 to December 2015) and phase 2 (from January 2016 to December 2020). We compared the parameters between patients treated in phases 1 and 2.

### Statistical analysis

Differences in clinicopathological characteristics were compared using Student's t-test, chi-square test, or Fisher's exact test. Cox proportional hazard regression analysis was used to estimate hazard ratios (HRs) and 95% confidence intervals (CIs). Disease-free survival (DFS) was defined as the interval between the date of initial diagnosis and the date of recurrence or the last follow-up. Overall survival (OS) was defined as the interval between the date of initial diagnosis and the date of cancer-related death or last follow-up. The Kaplan-Meier method with log-rank tests was used to compare DFS and OS between the two groups. All statistical analyses were performed using SPSS statistical software (version 25.0; SPSS Inc., Chicago, IL, USA). Statistical significance was set at P < 0.05.

## Results

In total, 142 patients were included in the final analysis. Of these, 73 and 69 patients underwent surgery in phase 1 (51.4%) and phase 2 (48.6%), respectively. Clinicopathological characteristics of the patients are presented in Table 1. The two groups showed no significant differences in age, stage, histological subtype, rate of preoperative conization, tumor size, or rate of lymphovascular space invasion (LVSI). In phase 2, a significantly higher percentage of patients underwent robotic surgery (4.1% vs. 43.5%, p = 0.001). Surgeons A and B performed similar numbers of surgeries during phases 1 and 2 (62 vs. 80 cases, p = 0.612). The median follow-up time was significantly shorter in phase 2 (phase 1, 86 months; phase 2, 35 months; p = 0.001). Twelve recurrences (12/142, 8.5%) were observed in the cohort at the time of analysis (Table 2): ten (13.7%) in phase 1 and two (2.9%) in phase 2. The recurrence rate was significantly higher in phase 1 (p = 0.021). Of the two recurrent cases in phase 2, one each involved LRH and RRH. The entire cohort involved four (2.8%) cancer-related deaths, all of which occurred in phase 1 (5.5%).

The sites of recurrence are listed in Table 2. In the phase 2 group, one case occurred in the pelvic lymph node and the other occurred on the surface of the spleen. In the phase 1 group, seven (70%) of the ten recurrences were locoregional (3 cases in the stump; 4 cases in the pelvic lymph node).

**Figure 1:**
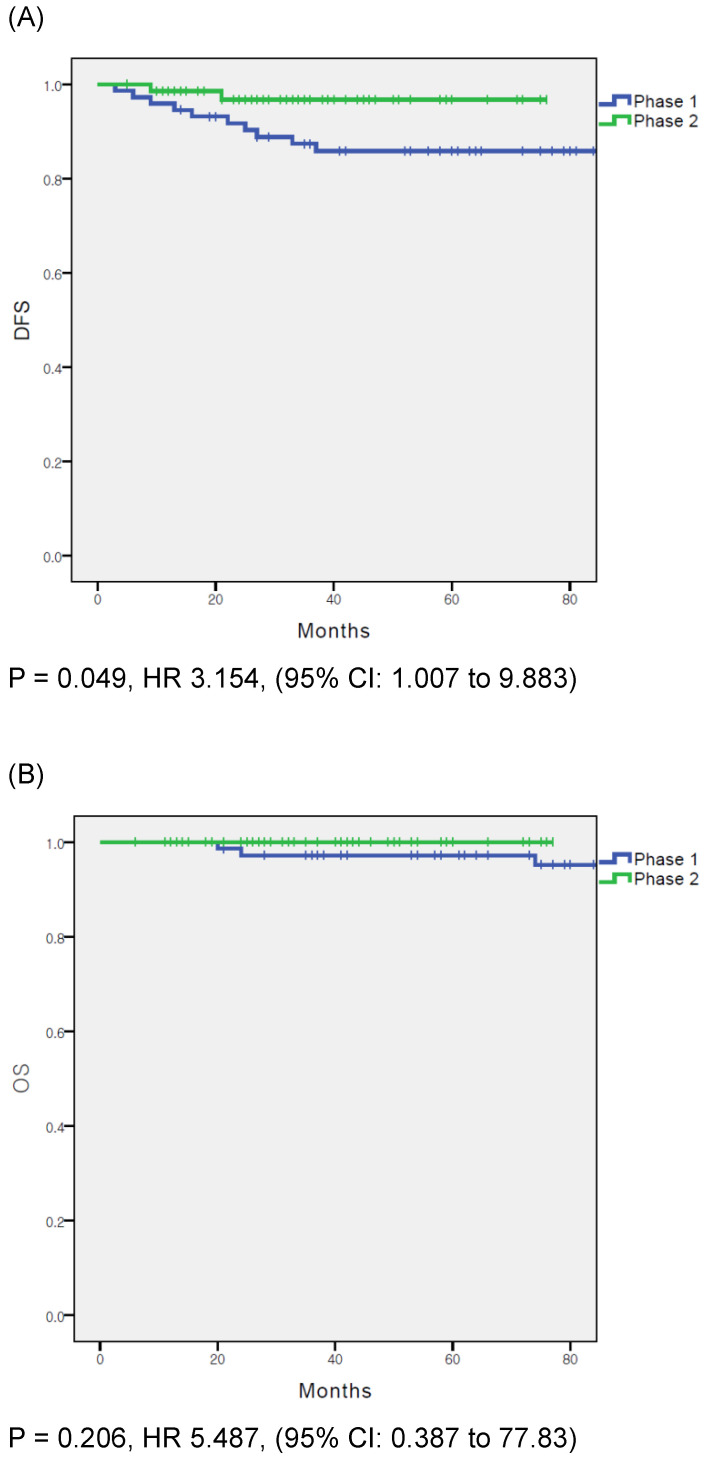
** (A)** disease free survival in entire cohort. **(B)** overall survival in entire cohort.

In the overall population, DFS was significantly different between the phase 1 and phase 2 groups (p = 0.049); however, OS did not differ significantly between the groups (p = 0.206) (Fig. 1A and B). Cox proportional hazards regression analysis was used to evaluate the prognostic factors for recurrence (Table 3). In the multivariate analysis, surgical proficiency, represented by the phase of operation, was the only significant predictor of DFS (HR = 0.244, p = 0.042). The other factors were not statistically significant.

## Discussion

In this study, we divided the cohort according to the year of surgery into phases 1 and 2 and compared the survival outcomes in the two phases. Interestingly, patients who underwent MIS in phase 2 showed better DFS than those who underwent the procedure in phase 1. Thus, our findings indicate a positive effect of surgical proficiency in MIS for early-stage cervical cancer. These results are in concordance with those of previously reported studies 15-18, 22.

The results of the LACC trial were reported in 2018 9, and they contradicted the results of previous studies that showed favorable outcomes for MIS in cervical cancer 6-8, 23. However, the LACC trial was associated with some issues. The first was related to the size of the tumor. Tumor size is a known prognostic factor in cervical cancer, and is included in the Sedlis criteria 21. Tumor size < 2 cm is known to indicate a low risk and is accepted for fertility-sparing and less radical surgery. After the LACC trial was published, many studies reported that tumor size < 2 cm was not related to inferior MIS outcomes 24-26. Other studies have reported that MIS is associated with inferior outcomes in women with tumors ≥ 2 cm 27, 28. The surgical approach used for MIS is another prognostic factor. In the LACC trial, only 16% of study participants underwent RRH. This does not reflect the current practice patterns in the United States or Europe. Moreover, some studies reported comparable outcomes between RRH and open surgery in early-stage cervical cancer 29-31. Third, favorable outcomes were observed after preoperative conization. Casarin et al. reported that preoperative conization had a protective role in patients with stage IB1 tumors 32. Similarly, the SUCCOR cone study found that patients who underwent conization before radical hysterectomy showed a significantly lower risk of relapse and death 33. Fourth, the surgical procedure can cause tumor breakdown, spillage, and dissemination. In the SUCCOR study, protective vaginal closure and avoiding the use of a uterine manipulator during MIS yielded similar outcomes to open surgery 34. Finally, the surgical proficiency of the surgeon is a factor that requires consideration. The LACC trial design included surgeons who could submit data from only 10 MIS cases and two unedited videos. However, several other studies have reported that more MIS cases are required to achieve surgical proficiency. Baeton et al. reported 61 cases, and Pedone et al. reported 19 17, 35. Park et al. argued that more than 40-50 cases are required for surgical proficiency 14. Thus, the requirements of the LACC trial design could be considered insufficient.

In our study, favorable outcomes were observed in the later phase of the study. The recurrence rate in the phase 1 group in our study (13.7%) was similar to that in the MIS group in the LACC trial (8.5%), and the recurrence rate in the phase 2 group in our study (2.9%) was similar to that in the open group in the LACC trial (2.2%). Comparisons of different surgeons and surgical approaches showed no statistically significant differences. In our institution, LRH was first introduced in 2004, and a robotic platform was adopted in 2014. Thus, surgical proficiency had been already achieved when RRH was initiated. These findings indicate the importance of surgical proficiency and show that favorable outcomes can be obtained using the MIS technique, irrespective of the surgical approach.

The strength of this study is that it was conducted at a single institution, minimizing the effects of differences between centers. Although this was a single-institution study, the sample size was comparable to that in previous studies 14-16. In addition, we compared the outcomes between the surgeons, which showed no statistically significant differences. This finding highlights the importance of surgical proficiency. However, this study had several limitations. First, due to the retrospective nature of the study, there may have been inevitable issues such as selection bias. In addition, missing data may have affected the data analysis. Second, although the sample size was comparable to that of other studies, it may have been insufficient to properly compare the clinical outcomes. Third, the actual radicality of the surgery was impossible to compare. Fourth, the phase 2 group showed a significantly shorter follow-up time. The favorable outcomes in the phase 2 group may be underestimated due to the shorter follow-up period, potentially overestimating the effect of proficiency.

Fifth, despite the importance of the frailty assessment of gynecological oncological patients, the frailty of the patients was not considered 36. Finally, perioperative and postoperative morbidities according to the surgical approach was not evaluated. However, Bogani et al. reported that the burden of treatment-related morbidity in early-stage cervical cancer patients after the publication of the LACC trial remained stable 37.

In conclusion, our study demonstrated that the early phase of MIS yielded inferior outcomes to those in the later phase. These findings suggest that surgical proficiency in MIS is a significant factor associated with the outcomes in early-stage cervical cancer. More favorable outcomes can be obtained after operating on a certain number of MIS cases. Further large-scale randomized controlled trials and clinical studies on sufficient surgical competence are required.

## Figures and Tables

**Table 1:** Clinicopathological characteristics of patients (n=142).

	Phase 1 (n = 73, %)	Phase 2 (n = 69, %)	p value
Age, years			
Mean ± SD	51.2 ± 12.2	51.6 ± 9.2	0.837
Surgical approach			< 0.001^*^
LRH	70 (95.9)	39 (56.5)	
RRH	3 (4.1)	30 (43.5)	
Surgeon			0.612
A	30 (41.1)	32 (46.4)	
B	43 (58.9)	37 (53.6)	
FIGO stage			0.368
IB1	59 (80.8)	60 (87.0)	
IB2	14 (19.2)	9 (13.0)	
Histology			0.172
SCC	59 (80.8)	48 (69.6)	
ACC	11 (15.1)	19 (27.5)	
ASC	3 (4.1)	2 (2.9)	
Preoperative conization	31 (42.5)	37 (53.6)	0.239
Tumor size (cm)			0.348
Mean ± SD	1.51 ± 0.75	1.39 ± 0.65	
LVSI	14 (19.2)	9 (13.0)	0.368
Adjuvant RT	25 (34.2)	15 (21.7)	0.135
Recurrences	10 (13.7)	2 (2.9)	0.021^*^
Deaths	4 (5.5)	0 (0)	0.120
Follow-up time (months)	86	35	< 0.001^*^
Median, range	14 - 154	6 - 77	

SD, standard deviation; LRH, conventional laparoscopic radical hysterectomy; RRH, robot-assisted laparoscopic radical hysterectomy; FIGO, International Federation of Gynecology and Obstetrics; SCC, squamous cell carcinoma; ACC, adenocarcinoma; ASC, adenosquamous carcinoma; LVSI, lymphovascular space invasion; RT, radiation therapy.

**Table 2:** Sites of disease recurrence.

	Phase 1 (n = 73, %)	Phase 2 (n = 69, %)	p value
Recurrence	10 (13.7)	2 (2.9)	0.021^*^
Site of recurrence, total Stump	10 3 (30.0)	2 0	0.745
Pelvic lymph node	4 (40.0)	1 (50)	
Lung	2 (20.0)	0	
Spleen	0	1 (50)	
Peritoneum	1 (10.0)	0	

**Table 3:** Multivariate analysis of factors correlated with disease free survival (n = 142).

Characteristics	Multivariate analysis		
	OR	95% CI	p value
Age, years	1.021	0.969 - 1.021	0.431
FIGO stage			
IB1	1 (Ref)	-	-
IB2	1.803	0.484 - 6.722	0.380
Histology	1 (Ref)	-	-
SCC	1.651	0.432 - 6.308	0.464
ACC	2.935	0.315 - 27.322	0.344
ASC			
Surgical approach			
LRH	1 (Ref)	-	-
RRH	0.592	0.052 - 6.770	0.673
Surgeon			
A	1 (Ref)	-	-
B	1.031	0.321 - 3.312	0.959
Phase			
1	1 (Ref)		
2	0.244	0.053 - 1.117	0.042^*^
Preoperative conization			
No	1 (Ref)		
Yes	0.770	0.156 - 3.793	0.748
LVSI			
No	1 (Ref)	-	-
Yes	0.345	0.030 - 3.905	0.388
Adjuvant treatment			
None	1 (Ref)	-	-
RT	1.193	0.230 - 6.179	0.834

OR, odds ratio; CI, confidence interval; Ref, reference; FIGO, International Federation of Gynecology and Obstetrics; SCC, squamous cell carcinoma; ACC, adenocarcinoma; ASC, adenosquamous carcinoma; LRH, conventional laparoscopic radical hysterectomy; RRH, robot-assisted laparoscopic radical hysterectomy; LVSI, lymphovascular space invasion; RT, radiation therapy.
